# Association between thyroid cysts and hypertension by atherosclerosis status: a cross-sectional study

**DOI:** 10.1038/s41598-021-92970-x

**Published:** 2021-07-06

**Authors:** Yuji Shimizu, Shin-Ya Kawashiri, Yuko Noguchi, Yasuhiro Nagata, Takahiro Maeda, Naomi Hayashida

**Affiliations:** 1 Department of Community Medicine, Nagasaki University Graduate School of Biomedical Sciences, Nagasaki-shi, Sakamoto 1-12-4, Nagasaki, 852-8523 Japan; 2 Department of Cardiovascular Disease Prevention, Osaka Center for Cancer and Cardiovascular Diseases Prevention, Osaka, Japan; 3 Center for Comprehensive Community Care Education, Nagasaki University Graduate School of Biomedical Sciences, Nagasaki, Japan; 4 Department of General Medicine, Nagasaki University Graduate School of Biomedical Sciences, Nagasaki, Japan; 5 Division of Promotion of Collaborative Research on Radiation and Environment Health Effects, Atomic Bomb Disease Institute, Nagasaki University, Nagasaki, Japan

**Keywords:** Biomarkers, Endocrinology, Medical research, Risk factors

## Abstract

Our recent studies indicate that thyroid cysts have clinical implications. Thyroid cysts could have a positive effect on the supply of thyroid hormones. Both hyperthyroidism and hypothyroidism cause hypertension. Hypothyroidism, but not hyperthyroidism, is a risk factor for atherosclerosis. Therefore, thyroid cysts could be associated with hypertension, and atherosclerosis might influence the association between thyroid cysts and hypertension. To evaluate the clinical significance of thyroid cysts, a cross-sectional study was conducted with 1801 Japanese aged 40–74 years. Thyroid cysts were significantly positively associated with hypertension in participants without atherosclerosis. However, there was a significant inverse association in those with atherosclerosis. The potential confounding factor adjusted odd ratios and 95% confidence intervals (95% CIs) were 1.49 (95% CI 1.17–1.90) for participants without atherosclerosis and 0.49 (95% CI 0.24–0.98) for those with atherosclerosis. The present study demonstrates that thyroid cysts have clinical implications because thyroid cysts support thyroid hormone activity. Our findings provide sufficient evidence to develop a risk assessment for hypertension for the general population, even though further research is required.

## Introduction

Although thyroid cysts are generally regarded to be clinically insignificant, our recent studies indicate that thyroid cysts might have clinical effects on the activity of thyroid hormones^1–3^. Anti-thyroid peroxidase antibody (TPO-Ab) is a known autoimmune antibody. The absence of thyroid cysts has been shown to be positively associated with TPO-Ab^1^. Thyroid disorder can lead to hypertension^4^. Therefore, among participants with normal thyroid function, participants with TPO-Ab (+) might have latent thyroid damage. TPO-Ab (+) status has been shown to be associated with hypertension-related latent thyroid damage. TPO-Ab (+) status is positively associated with subclinical hypothyroidism and hypertension, but not with subclinical hypothyroidism without hypertension^2^.

In addition, this previous study also showed that the presence of thyroid cysts might influence the association between subclinical hypothyroidism and hypertension; subclinical hypothyroidism is positively associated with hypertension among participants without thyroid cysts but not participants with thyroid cysts^2^. These associations could indicate that thyroid cysts can prevent hypothyroidism-related hypertension. Therefore, thyroid cysts could help maintain the latent function of thyroid hormone activity.

Both hyperthyroidism and hypothyroidism can lead to hypertension^4^. Therefore, the presence of thyroid cysts could be associated with hypertension by enhancing the effect of hyperthyroidism and reducing the effect of hypothyroidism in the general population.

In addition, lower levels of the thyroid hormone triiodothyronine (T3) were demonstrated to be associated with the progression of atherosclerosis in coronary arteries^5^. Therefore, an analysis of participants with atherosclerosis might strengthen the influence of decreased thyroid hormone activity. Meanwhile, an analysis of participants without atherosclerosis might strengthen the influence of higher activity of thyroid hormones [T3 and thyroxine (T4)]. Since the normal range of TPO-Ab titers is associated with atherosclerosis among euthyroid individuals^6^, latent damage of the thyroid might also influence the progression of atherosclerosis.

Therefore, the clinical characteristics of thyroid cysts might influence atherosclerosis and hypertension. To evaluate the clinical significance of thyroid cysts, we conducted a cross-sectional study of 1722 Japanese aged 40–74 years who participated in an annual health check-up in 2014.

## Materials and methods

### Study population

The methods related to the present risk surveys, including thyroid function, have been described elsewhere^1–3^. Researchers ensured that prospective participants understood the objectives of the study. Written informed consent was obtained. This study was approved by the ethics committee of the Nagasaki University Graduate School of Biomedical Sciences (project registration number 14051404).

All of the study procedures involving human participants were performed in accordance with the ethical standards of the institution research committee and the 1964 Helsinki Declaration and later amendments for comparable ethical standards.

Study participants consisted of 1883 Japanese individuals aged 40–74 years from the town of Saza in western Japan. They underwent an annual medical check-up in 2014, as recommended by the Japanese government.

To avoid the influence of thyroid diseases, we excluded all participants with a history of thyroid disease (n = 60) and those without data on thyroid function. We excluded those with missing values for thyroid stimulating hormone (TSH), free T3, or free T4 (n = 17). We also excluded participants without data on body mass index (BMI) (n = 1), blood pressure (n = 1), smoking status (n = 2), and drinking status (n = 1).

Finally, 1801 participants with a mean age of 60.5 years [standard deviation (SD): 9.2; range 40–74] were analyzed in the present study.

### Data collection and laboratory measurements

Trained interviewers obtained information about clinical characteristics. Body weight and height were measured with an automatic body composition analyzer (BF-220; Tanita, Tokyo, Japan). Based on those measurements, BMI (kg/m^2^) was calculated. Resting systolic blood pressure (SBP) and diastolic blood pressure (DBP) were recorded. Hypertension was defined as SBP ≥ 140 mmHg, DBP ≥ 90 mmHg, or use of anti-hypertensive medication. Systolic hypertension was defined as SBP ≥ 140 mmHg while diastolic hypertension was defined as DBP ≥ 90 mmHg.

Fasting blood samples were collected. TSH, free T3, and free T4 were measured using the standard procedure at the LSI Medience Corporation (Tokyo, Japan). Hemoglobin A1c (HbA1c), triglycerides (TG), and high-density lipoprotein-cholesterol (HDLc) were measured using the standard procedure at SRL, Inc. (Tokyo, Japan).

Detection of thyroid cysts and evaluation of carotid intima-media thickness (CIMT) were performed by experienced technicians using a LOGIQ Book XP with a 10-MHz transducer (GE Healthcare, Milwaukee, WI, USA). In this study, thyroid cyst was defined as a structure with a maximum diameter of ≥ 2.0 mm containing no solid components. Maximum CIMT values for the left and right common carotid arteries were calculated with semi-automated digital edge-detection software (Intimascope; MediaCross, Tokyo, Japan) using a protocol that has been described in detail elsewhere^7^. Peak right and left CIMT measurements excluding plaques were calculated. The higher CIMT value was then used for analysis. Since a previous study reported normal CIMT as < 1.1 mm^8^, we defined atherosclerosis as CIMT ≥ 1.1 mm.

### Statistical analysis

Male gender, anti-hypertensive medication use, daily drinking, and current smoker status were presented as percentages. Other characteristics of the study population were expressed as means ± SD by thyroid cyst status. Since TSH had a skewed distribution, the characteristics of the study population were expressed as medians (interquartile range), followed by logarithmic transformation. Differences by thyroid cyst status were calculated.

Logistic regression was used to calculate odds ratios (ORs) and 95% confidence intervals (CIs) of hypertension and atherosclerosis in relation to free T3. Logistic regression was also used to calculate ORs and 95% CIs to identify possible associations between hypertension, systolic hypertension, diastolic hypertension, and thyroid cysts.

In addition, study participants were stratified by atherosclerosis status. Three adjustment models were used. The first model adjusted for sex and age only (Model 1). The second model (Model 2) adjusted for variables in Model 1 plus potential confounding factors that are well known cardiovascular risk factors, such as BMI (kg/m^2^), smoking status (never, former, and current), drinking status (non, often, and daily), HbA1c (%), TG (mg/dL), and HDLc (mg/dL). The third model (Model 3) adjusted for variables in Model 2 plus variables directly associated with thyroid function: TSH (μIU/mL), free T3 (pg/mL), and free T4 (ng/mL).

All statistical analyses were performed with SAS for Windows, version 9.4 (SAS Inc., Cary, NC, USA); *p* < 0.05 was regarded as statistically significant.

## Results

### Clinical characteristics of the study population by thyroid cyst status

Clinical characteristics of the study population by thyroid cyst status are shown in Table 1. The proportion of men among participants with thyroid cysts was significantly lower than the proportion among participants without thyroid cysts. Participants with thyroid cysts were significantly older and had significantly higher SBP than those without thyroid cysts.

**Table 1:** Characteristics of the study population.

	Thyroid cyst		*p*
	(−)	(+)	
No at risk	1213	588	
Men (%)	40.7	29.8	< 0.001
Age (year)	59.7 ± 9.5	62.2 ± 8.3	< 0.001
TSH (μIU/mL)	1.58 [1.10, 2.32]^a^	1.63 [1.08, 2.43]^a^	0.819^b^
Free T3 (pg/mL)	3.2 ± 0.3	3.1 ± 0.3	0.178
Free T4 (ng/dL)	1.2 ± 0.2	1.2 ± 0.2	0.155
BMI (kg/m^2^)	22.9 ± 3.4	22.6 ± 3.3	0.132
SBP (mmHg)	124 ± 17	126 ± 17	0.005
DBP (mmHg)	73 ± 11	74 ± 10	0.067
Anti-hypertensive medication (%)	28.2	32.8	0.044
Current smoker (%)	14.5	11.4	0.070
Daily drinker (%)	40.6	38.9	0.491
HbA1c (%)	5.6 ± 0.6	5.6 ± 0.6	0.253
TG (mg/dL)	109 ± 87	101 ± 60	0.051
HDLc (mg/dL)	60 ± 15	61 ± 15	0.062

Values are mean ± standard deviation.

*TSH* thyroid stimulating hormone, *T3* triiodothyronine, *T4* thyroxine, *BMI* Body mass index, *SBP* systolic blood pressure, *DBP* diastolic blood pressure, *HbA1c* hemoglobin A1c, *TG* triglycerides, *HDLc* HDL-cholesterol.

^a^Values are median [the first quartile, third quartile].^b^ Logarithmic transformation was used for evaluating *p*.

### Association between hypertension and thyroid cyst status among all participants

Table 2 shows the ORs for hypertension in relation to the presence of thyroid cysts among all participants. The *p* value for Model 1 was greater than 0.05, indicating no significant difference in hypertension between participants with and without thyroid cysts. However, after adjustment for known cardiovascular risk factors (Model 2), there was a statistically significant positive association between hypertension and the presence of thyroid cysts. Model 3, which also adjusted for thyroid hormone levels, also showed a significant association. The adjusted ORs for hypertension with respect to the presence of thyroid cysts were 1.23 (95% CI 0.99–1.53) for Model 1, 1.31 (95% CI 1.04–1.65) for Model 2, and 1.32 (95% CI 1.05–1.65) for Model 3.

**Table 2:** Association between the presence of thyroid cysts and hypertension.

	Thyroid cyst		*p*
	(−)	(+)	
Number of participants	1213	588	
Number of case (%)	449 (37.0)	261 (44.4)	
Model 1 OR (95% CI)	Ref	1.23 (0.99, 1.53)	0.067
Model 2 OR (95% CI)	Ref	1.31 (1.04, 1.65)	0.020
Model 3 OR (95% CI)	Ref	1.32 (1.05, 1.65)	0.019

Model 1: adjusted for sex and age. Model 2: + BMI, smoking status (never, former, current), drinking status (non, often, daily) TG, HDLc, HbA1c. Model 3: + TSH, free T3, free T4. Hypertension was defined as SBP ≥ 140 mmHg and/or DBP ≥ 90 mmHg and/or taking anti-hypertensive medication.

*OR* odds ratio, *CI* confidence interval, *Ref* reference, *BMI* Body mass index, *TG* triglycerides, *HDLc* HDL-cholesterol, *HbA1c* hemoglobin A1c, *TSH* thyroid stimulating hormone, *T3* triiodothyronine, *T4* thyroxine, *SBP* systolic blood pressure, *DBP* diastolic blood pressure.

### Association between hypertension and free triiodothyronine (T3) levels

A U-shaped association between hypertension and free T3 was observed, even though the analysis did not have sufficient power. With the middle quintile of free T3 (Q3) as the reference group, the crude OR for hypertension was 1.24 (95% CI 0.92–1.68) for the lowest level (Q1) and 1.21 (95% CI 0.89–1.64) for the highest level (Q5) (Table 3).

**Table 3:** Association between hypertension, atherosclerosis and free triiodothyronine (T3) levels.

	Free triiodothyronine (T3) levels					*p*
	Q 1 (low)	Q2	Q3	Q4	Q5 (high)	
Number of participants	376	350	348	381	346	
**Hypertension**						
Number of case (%)	159 (42.3)	130 (37.1)	129 (37.1)	148 (38.8)	144 (41.6)	
Crude model OR (95% CI)	1.24 (0.92, 1.68)	1.00 (0.74, 1.36)	Ref	1.08 (0.80, 1.46)	1.21 (0.89, 1.64)	0.985
**Atherosclerosis**						
Number of case (%)	51 (13.6)	31 (8.9)	30 (8.6)	35 (9.2)	27 (7.8)	
Crude model OR (95% CI)	Ref	0.62 (0.39, 0.99)	0.60 (0.37, 0.97)	0.65 (0.41, 1.02)	0.54 (0.33, 0.88)	0.021

Quintile values of free triiodothyronine (T3) for men and women are < 3.1 pg/mL and < 2.9 pg/mL for Q1, 3.1 pg/mL and 2.9–3.0 pg/mL for Q2, 3.2–3.3 pg/mL and 3.1 pg/mL for Q3, 3.4–3.5 pg/mL and 3.2–3.3 pg/mL for Q4, and 3.6 pg/mL ≤ and 3.4 pg/mL ≤ for Q5. Hypertension was defined as SBP ≥ 140 mmHg and/or DBP ≥ 90 mmHg and/or taking anti-hypertensive medication. Atherosclerosis was defined as CIMT ≥ 1.1 mm.

*OR* odds ratio, *CI* confidence interval, *Ref* reference, *SBP* systolic blood pressure, *DBP* diastolic blood pressure, *CIMT* carotid intima-media thickness.*OR* odds ratio, *CI* confidence interval, *Ref* reference, *SBP* systolic blood pressure, *DBP* diastolic blood pressure, *CIMT* carotid intima-media thickness.

### Association between atherosclerosis and free triiodothyronine (T3) levels

In the crude model, an inverse association between atherosclerosis and free T3 was observed. With the lowest quintile of free T3 (Q1) as the reference group, the crude OR for atherosclerosis was 0.62 (95% CI 0.39–0.99) for Q2, 0.60 (95% CI 0.37–0.97) for Q3, 0.65 (95% CI 0.41–1.02) for Q4, and 0.54 (95% CI 0.33–0.88) for Q5 (Table 3).

### Association between hypertension and thyroid cyst status, stratified by atherosclerosis status

Table 4 shows the association between hypertension and the presence of thyroid cysts after stratifying by the presence of atherosclerosis. Among participants without atherosclerosis, the presence of thyroid cysts had a statistically significant positive association with hypertension (Model 1: OR 1.34; 95% CI 1.06–1.69; *p* = 0.015). This association remained unchanged after further adjustment for known cardiovascular risk factors and thyroid hormone levels (Model 3: adjusted OR 1.49; 95% CI 1.17–1.90; *p* = 0.001).

**Table 4:** Association between the presence of thyroid cysts and hypertension by the existence of atherosclerosis.

	Atherosclerosis (−)			Atherosclerosis (+)			Interaction
	Thyroid cyst		*p*	Thyroid cyst		*p*	
	(−)	(+)		(−)	(+)		
Number of participants	1109	518		104	70		
Number of case (%)	388 (35.0)	229 (44.2)		61 (58.7)	32 (45.7)		
Model 1 OR (95% CI)	Ref	1.34 (1.06, 1.69)	0.015	Ref	0.60 (0.32, 1.14)	0.119	0.020
Model 2 OR (95% CI)	Ref	1.49 (1.16, 1.90)	0.002	Ref	0.45 (0.22, 0.94)	0.033	0.002
Model 3 OR (95% CI)	Ref	1.49 (1.17, 1.90)	0.001	Ref	0.49 (0.24, 0.98)	0.043	0.002

Model 1: adjusted for sex and age. Model 2: + BMI, smoking status (never, former, current), drinking status (non, often, daily) TG, HDLc, HbA1c. Model 3: + TSH, free T3, free T4. Hypertension was defined as SBP ≥ 140 mmHg and/or DBP ≥ 90 mmHg and/or taking anti-hypertensive medication. Atherosclerosis was defined atherosclerosis as CIMT ≥ 1.1 mm.

*OR* odds ratio, *CI* confidence interval, *Ref* reference, *BMI* Body mass index, *TG* triglycerides, *HDLc* HDL-cholesterol, *HbA1c* hemoglobin A1c, *TSH* thyroid stimulating hormone, *T3* triiodothyronine, *T4* thyroxine, *SBP* systolic blood pressure, *DBP* diastolic blood pressure, *CIMT* carotid intima-media thickness.

Regarding atherosclerosis, Model 1 showed a non-significant inverse tendency (OR 0.60; 95% CI 0.32–1.14; *p* = 0.119). After further adjustment for known cardiovascular risk factors (Model 2), the association was statistically significant (adjusted OR 0.45; 95% CI 0.22–0.94; *p* = 0.033). This significant association remained unchanged after further adjustment for thyroid hormone levels (adjusted OR 0.49; 95% CI 0.24–0.98; *p* = 0.043).

The interaction between thyroid cysts and atherosclerosis was associated with hypertension. The adjusted *p* value for the effect of the interaction was *p* = 0.020 for Model 1, *p* = 0.002 for Model 2, and *p* = 0.002 for Model 3 (Table 4).

For sensitivity analysis, sex-specific associations between hypertension and thyroid cyst status and stratified by atherosclerosis status were evaluated in an age-adjusted model. Although power was insufficient due to the limited number of participants, essentially the same associations were observed for men and women. Among participants without atherosclerosis, positive tendencies were observed in men (n = 581) (adjusted OR 1.44; 95% CI 0.96–2.16; *p* = 0.077) and women (n = 1046) (adjusted OR 1.29; 95% CI 0.97–1.72; *p* = 0.081). Among participants with atherosclerosis, inverse tendencies were observed in men (n = 88) (adjusted OR 0.67; 95% CI 0.27–1.68; *p* = 0.393) and women (n = 86) (adjusted OR 0.57; 95% CI 0.23–1.41; *p* = 0.225).

### Association between hypertension and thyroid cyst status, stratified by atherosclerosis status in participants not taking anti-hypertensive medications

Among participants without atherosclerosis, there was a statistically significant positive association between the presence of thyroid cysts and systolic hypertension, but not with diastolic hypertension. Meanwhile, among participants with atherosclerosis, non-statistically significant inverse associations were observed for both systolic and diastolic hypertension (Table 5).

**Table 5:** Association between the presence of thyroid cysts and hypertension by the existence of atherosclerosis in participants without taking anti-hypertensive medication.

	Atherosclerosis (−)			Atherosclerosis (+)			Interaction
	Thyroid cyst		*p*	Thyroid cyst		*p*	
	(−)	(+)		(−)	(+)		
**Systolic hypertension**							
Number of participants	812	349		59	46		
Number of case (%)	83 (10.2)	57 (16.3)		14 (23.7)	8 (17.4)		
Model 1 OR (95% CI)	Ref	1.61 (1.10, 2.36)	0.014	Ref	0.65 (0.24, 1.73)	0.385	0.109
Model 2 OR (95% CI)	Ref	1.70 (1.15, 2.52)	0.008	Ref	0.46 (0.14, 1.54)	0.207	0.0497
Model 3 OR (95% CI)	Ref	1.71 (1.15, 2.53)	0.008	Ref	0.36 (0.10, 1.37)	0.135	0.044
**Diastolic hypertension**							
Number of participants	812	349		59	46		
Number of case (%)	46 (5.7)	20 (5.7)		5 (8.5)	3 (6.5)		
Model 1 OR (95% CI)	Ref	1.07 (0.61, 1.86)	0.825	Ref	0.88 (0.19, 4.08)	0.874	0.711
Model 2 OR (95% CI)	Ref	1.11 (0.63, 1.96)	0.712	Ref	0.55 (0.09, 3.28)	0.509	0.450
Model 3 OR (95% CI)	Ref	1.13 (0.64, 1.99)	0.683	Ref	0.58 (0.09, 3.96)	0.577	0.397

Model 1: adjusted for sex and age. Model 2: + BMI, smoking status (never, former, current), drinking status (non, often, daily) TG, HDLc, HbA1c. Model 3: + TSH, free T3, free T4. Systolic hypertension was defined as SBP ≥ 140 mmHg while diastolic hypertension was defined as DBP ≥ 90 mmHg. Atherosclerosis was defined atherosclerosis as CIMT ≥ 1.1 mm.

*OR* odds ratio, *CI* confidence interval, *Ref* reference, *BMI* Body mass index, *TG* triglycerides, *HDLc* HDL-cholesterol, *HbA1c* hemoglobin A1c, *TSH* thyroid stimulating hormone, *T3* triiodothyronine, *T4* thyroxine, *SBP* systolic blood pressure, *DBP* diastolic blood pressure, *CIMT* carotid intima-media thickness.

## Discussion

The major finding of our present study is that the presence of atherosclerosis could affect the association between the presence of thyroid cysts and hypertension in the general population.

Previously, our study with 1432 euthyroid Japanese individuals revealed a significant inverse association between TPO-Abs and thyroid cysts^1^. Since TPO-Ab is a known autoimmune antibody that causes autoimmune thyroiditis, absence of thyroid cysts among euthyroid individuals might indicate the presence of latent thyroid damage. TPO-Ab (+) status is associated with subclinical hypothyroidism and hypertension, but not with subclinical hypothyroidism without hypertension^2^. Furthermore, the normal range of TPO-Ab titers is associated with atherosclerosis among euthyroid participants^6^. Atherosclerosis and hypertension are known to be closely connected^9^. Therefore, even in individuals normal thyroid function, there could be an unknown connection among the presence of thyroid cysts, hypertension, and atherosclerosis.

In the present study, we found further evidence that the presence of thyroid cysts is positively associated with hypertension in participants without atherosclerosis and inversely associated with hypertension in those with atherosclerosis.

**Figure 1:**
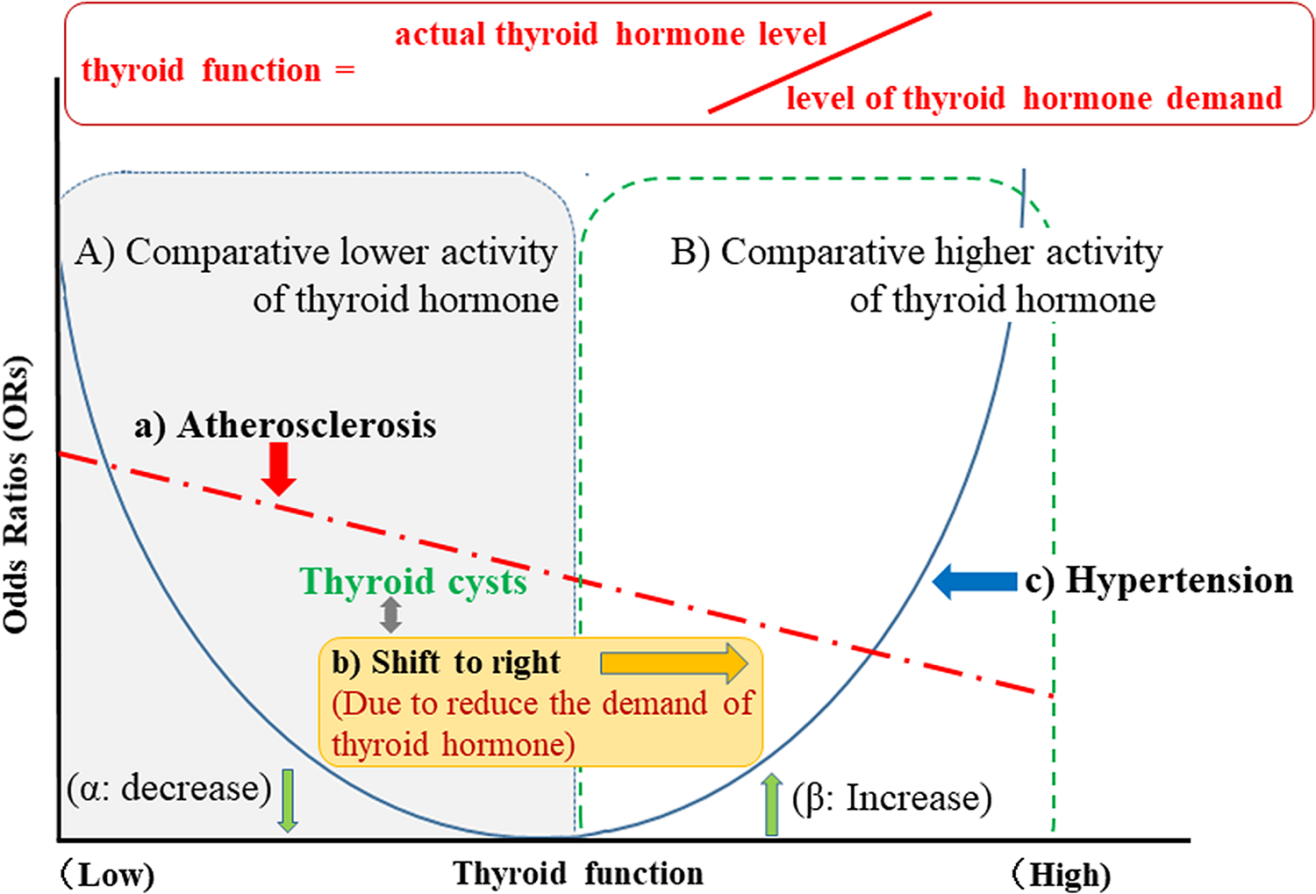
Association between hypertension, atherosclerosis and thyroid function (This figure was created by using Microsoft PowerPoint 2016; https://www.microsoft.com/ja-jp/microsoft-365/powerpoint).

Figure 1 shows the association between hypertension, atherosclerosis, and thyroid function.

Thyroglobulin is a crucial factor in the synthesis of T3 and T4^10^. Thyroglobulin is abundant in cyst fluid^11^. Therefore, thyroid cysts might have a beneficial influence on synthesis thyroid hormones.

School age is an important period of physical growth. Lower thyroid function, as in congenital hypothyroidism, is known to be associated with slow growth. The number of thyroid cysts was previously shown to be associated with more growth in school-age children^12^. This finding supports our hypothesis that the presence of thyroid cysts indicates a higher capacity for synthesizing and transporting thyroid hormones (T3 and T4).

In addition, our previous study found that subclinical hypothyroidism is positively associated with hypertension among participants without thyroid cysts but not among participants with thyroid cysts^2^. Since hypothyroidism is reported to be associated with hypertension^13^, the presence of thyroid cyst might have a beneficial effect on the activation of thyroid hormones.

However, in this study, independent from known cardiovascular risk factors, thyroid hormones (free T3 and free T4), and TSH, the presence of thyroid cysts was significantly positively associated with hypertension (Table 2). Therefore, serum concentrations of thyroid hormones and TSH are not efficient tools for evaluating the function of thyroid cysts.

Our study population included adults aged 40–74 years. We found that participants with thyroid cysts were significantly older than those without thyroid cysts (Table 1). A previous study reported that decreased thyroid function might lead to extended longevity^14^. Therefore, the aging process might decrease the demand for thyroid hormone activity, which results in relatively higher thyroid hormone activity in elderly participants with the same thyroid hormone levels as younger participants. The following equation could be established: thyroid function = actual thyroid hormone level/level of thyroid hormone demand. Participants with comparatively higher thyroid hormone activity (thyroid function) might have formed thyroid cysts to store abundant thyroglobulin. In this study, no significant differences in TSH, free T3, or free T4 levels were observed between patients with and without thyroid cysts (Table 1). Although evaluation of thyroid function without information on the level of demand is not accurate, serum concentrations of free T3 could indicate thyroid function to same extent.

Since hyperthyroidism and hypothyroidism can lead to hypertension^4^, we found a U-shaped association between free T3 levels and hypertension in the crude model (Table 3; Fig. 1c) despite insufficient power.

Since subclinical hypothyroidism is positively associated with hypertension among participants without thyroid cysts but not among participants with thyroid cysts^2^, the presence of thyroid cysts might increase thyroid function in participants with lower thyroid hormone activity, partly by decreasing the demands for thyroid hormones, resulting in a lower risk of hypertension. As shown in Fig. 1b, decreased demands for thyroid hormones associated with thyroid cysts increase thyroid function and shift the axis of thyroid function to the right. This right shift reduces the risk of hypertension among participants with comparative lower thyroid hormone activity (Fig. 1α) while increasing the risk of hypertension among participants with comparative higher thyroid hormone activity (Fig. 1β).

Furthermore, although both hyperthyroidism and hypothyroidism affect the cardiovascular system, hypothyroidism is known to be associated with the progression of atherosclerosis^15^. In the present study, the crude model revealed an inverse association between free T3 and atherosclerosis (Table 3; Fig. 1a). Since the prevalence of atherosclerosis is higher among participants with lower thyroid hormone activity (Fig. 1A) than those with higher thyroid hormone activity (Fig. 1B), the analysis of participants with atherosclerosis emphasizes the influence of lower thyroid hormone activity. Participants with thyroid cysts had higher thyroid function due to a reduced demand for thyroid hormones (Fig. 1b). Among participants with thyroid cysts, the risk of hypertension was lower among those with atherosclerosis (Table 4; Fig. 1α) whereas the risk of hypertension was higher for those without atherosclerosis (Table 4; Fig. 1β).

**Figure 2:**
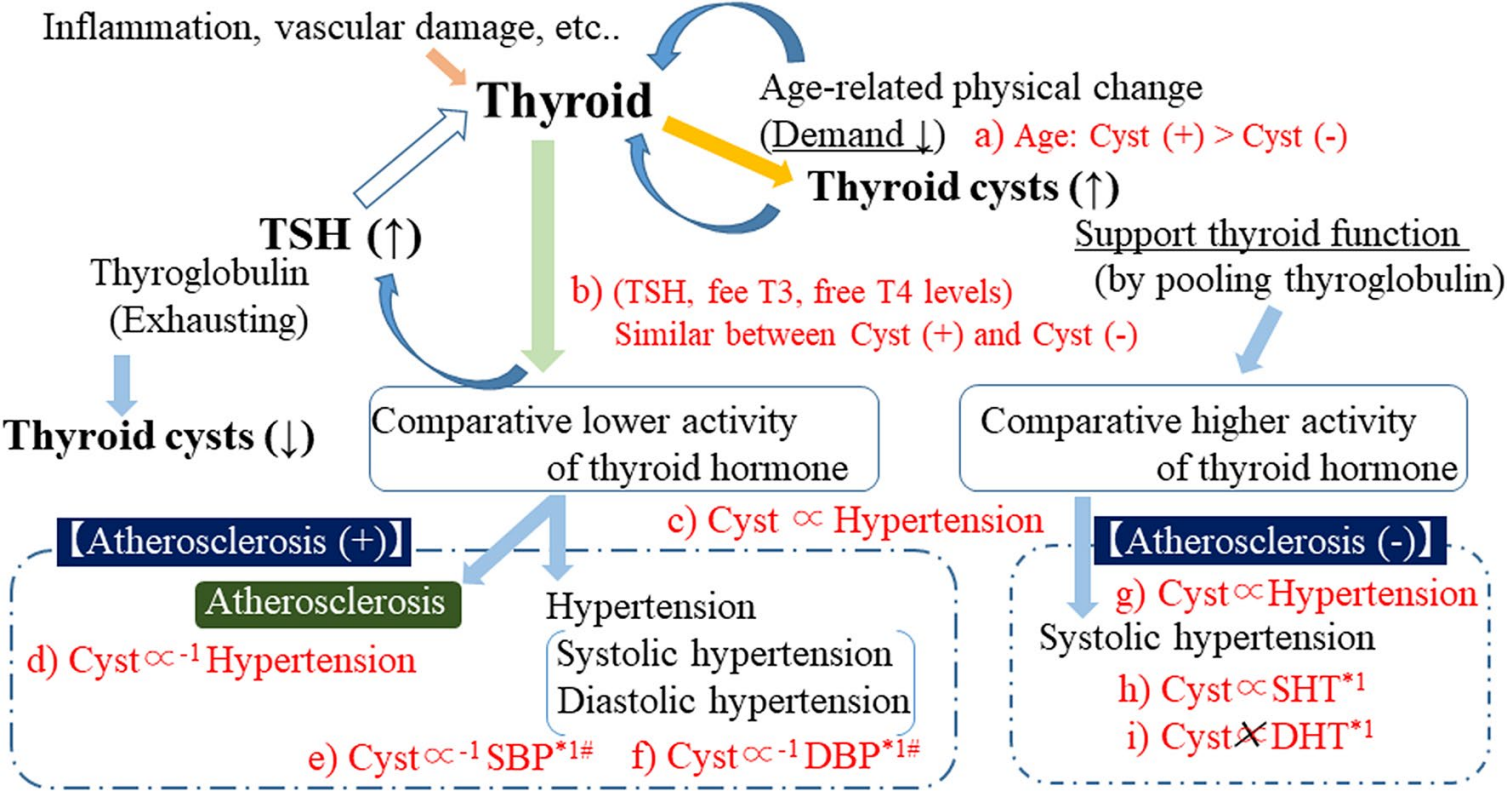
Potential mechanisms for the present results. Association shown in red (**a**–**i**) were observed in the present study. *TSH* thyroid stimulating hormone, *free T3* free triiodothyronine, *Cyst* thyroid cysts, *SHT* systolic hypertension, *DHT* diastolic hypertension. *1: Observed among participants without taking anti-hypertensive medication. #: The statistical power could not reach significant level. (This figure was created by using Microsoft PowerPoint 2016; https://www.microsoft.com/ja-jp/microsoft-365/powerpoint).

Figure 2 shows the potential mechanisms for the present results.

Decreased demand for thyroid hormones associated with the process of aging^14^ might lead to the formation of thyroid cysts that pool thyroglobulin^11^ (Table 1; Fig. 2a). Even though participants with and without thyroid hormone had similar serum levels of thyroid hormones, participants with thyroid cysts might have higher levels of thyroid hormone activity because of the influence of age-related reductions in the demand for thyroid hormone (Table 1; Fig. 2b).

Hyperthyroidism is a common cause of isolated systolic hypertension^16^. The presence of thyroid cysts was shown to be positively associated with systolic hypertension but not with diastolic hypertension^3^. These studies also support our hypothesis that participants with thyroid cysts could have higher thyroid hormone activity than participants without thyroid cysts. In this study, we found a significant positive association between thyroid cysts and hypertension (Table 2; Figs. 1b, 2c), possibly indicating that participants with thyroid cysts have higher thyroid hormone activity than participants without thyroid cysts.

Since hypothyroidism is known to be associated with the progression of atherosclerosis^15^, the influence of lower thyroid hormone activity in participants with atherosclerosis was stronger than in participants without atherosclerosis. Therefore, in our present study, we found significant inverse associations between the presence of thyroid cysts and hypertension in participants with atherosclerosis (Table 4; Figs. 1α, 2d). Atherosclerosis could enhance the beneficial effect of preventing hypothyroidism that thyroid cysts might possess.

Further analyses performed among participants with atherosclerosis who were not taking antihypertensive medications showed thyroid cysts have an inverse non-statistically significant association with both systolic and diastolic hypertension (Table 5; Fig. 2e,f). Further investigation with a larger study population is necessary. However, the present study suggests that the presence of thyroid cysts could have a beneficial influence on preventing both systolic and diastolic hypertension among participant with atherosclerosis by supporting thyroid function.

However, we also observed a significant positive association between hypertension and the presence of thyroid cysts in participants without atherosclerosis (Table 4; Figs. 1β, 2g). T3 plays an important role in reducing systemic vascular resistance^17,18^, which lowers diastolic blood pressure. However, T3 is also reported to enhance the force of myocardial contractions^19^, which elevates systolic blood pressure. Therefore, increased T3 activity could be associated with systolic hypertension but not with diastolic hypertension. Indeed, a previous study reported hyperthyroidism as a common cause of isolated systolic hypertension^16^. The presence of thyroid cysts might, to a certain extent, indicate that the thyroid can produce a sufficient amount of thyroid hormones. Therefore, the production of thyroid hormones is easily accelerated in participants with thyroid cysts. In our present study, significant positive associations between systolic hypertension and thyroid cysts were observed among participants without atherosclerosis who were not taking antihypertensive medications (Table 5; Fig. 2h). However, there were no significant associations between diastolic hypertension and the presence of thyroid cysts (Table 5; Fig. 2i).

Unlike the results of a previous general population study, present single population study pointed out completely different associations between participants with and without thyroid cysts. This study provided novel evidence that the same factor has both favorable and unfavorable characteristics for health maintenance. Moreover, the clinical status of the participants might determine these characteristics, as in our previous study^20,21^. Therefore, this study provided new insights into mechanisms related to thyroid hormones.

The clinical perspective of our present study is that even though thyroid cysts are generally regarded to be less clinically important, data on their presence and atherosclerosis could be helpful in evaluating health risks (e.g., hypertension) in daily clinical practice. Furthermore, this study also revealed the necessity of evaluating an individual’s thyroid hormone demands when evaluating individual thyroid function.

Our present study has certain limitations. First, we evaluated the presence or absence of thyroid cysts, but the number and size of the cysts might also be important factors. Although cyst fluid was rich in thyroglobulin, the presence of anti-thyroglobulin antibodies reduces thyroglobulin levels dramatically. It might act as a strong confounding factor in our present analysis. However, because of the limited volume of each blood sample, we were not able to evaluate the influence of anti-thyroglobulin antibodies. Therefore, it is of great importance to conduct further investigations focusing on the number and size of cysts, as well as the presence of anti-thyroglobulin antibodies. Although the metabolism of thyroid hormones in participants with thyroid cysts who do not have atherosclerosis might be accelerated, we could not evaluate this relationship. Finally, as this study is cross-sectional by nature, no causal relationship could be established.

## Conclusion

In conclusion, in the general population, the presence of thyroid cysts was positively associated with hypertension in participants without atherosclerosis and inversely associated with hypertension in those with atherosclerosis. Although it is necessary to carry out further investigation, our present findings could sufficiently inform the development of a method to assess the risk of hypertension for the general population.

## Data Availability

We cannot publicly provide individual data due to participant privacy, according to ethical guidelines in Japan. Additionally, the informed consent was obtained does not include a provision for publicity sharing data. Qualifying researchers may apply to access a minimal dataset by contacting Prof Naomi Hayashida, Principal Investigator, Division of Promotion of Collaborative Research on Radiation and Environment Health Effects, Atomic Bomb Disease Institute, Nagasaki University, Nagasaki, Japan at naomin@nagasaki-u.ac.jp. Or, please contact the office of data management at ritouken@vc.fctv-net.jp. Information for where data request is also available at https://www.genken.nagasaki-u.ac.jp/dscr/message/ and http://www.med.nagasaki-u.ac.jp/cm/.

## References

[1] Shimizu Y (2020). Anti-thyroid peroxidase antibody and thyroid cysts among the general Japanese population: a cross-sectional study. Environ. Health Prev. Med..

[2] Shimizu Y (2020). Anti-thyroid peroxidase antibody and subclinical hypothyroidism in relation to hypertension and thyroid cysts. PLoS ONE.

[3] Shimizu Y (2020). Associations between thyroid-stimulating hormone and hypertension according to thyroid cyst status in the general population: a cross sectional study. Environ. Health Prev. Med..

[4] Berta E (2019). Hypertension in thyroid disorders. Front. Endocrinol. (Lausanne).

[5] Barth JD (1987). Progression and regression of human coronary atherosclerosis. The role of lipoproteins, lipases and thyroid hormones in coronary lesion growth. Atherosclerosis.

[6] Shimizu Y (2020). Normal range of anti-thyroid peroxidase antibody (TPO-Ab) and atherosclerosis among eu-thyroid population: A cross-sectional study. Medicine (Baltimore).

[7] Hara T (2006). Evaluation of clinical markers of atherosclerosis in young and elderly Japanese adults. Clin. Chem. Lab. Med..

[8] Kawamori R (1992). Prevalence of carotid atherosclerosis in diabetic patients. Ultrasound high-resolution B-mode imaging on carotid arteries. Diabetes Care.

[9] Shimizu Y (2017). Platelets and circulating CD34-positive cells as an indicator of the activity of the vicious cycle between hypertension and endothelial dysfunction in elderly Japanese men. Atherosclerosis.

[10] Citterio CE, Targovnik HM, Arvan P (2019). The role of thyroglobulin in thyroid hormonogenesis. Nat. Rev. Endocrinol..

[11] Salabè GB (1990). Identification of serum proteins, thyroglobulin and antithyroid antibodies in the fluid of thyroid cysts. Thyroidology.

[12] Hayashida N (2013). Investigation Committee for the Proportion of Thyroid Ultrasound Findings. Thyroid ultrasound findings in children from three Japanese prefectures: Aomori, Yamanashi and Nagasaki. PLoS ONE.

[13] Stabouli S, Papakatsika S, Kotsis V (2010). Hypothyroidism and hypertension. Expert Rev. Cardiovasc. Ther..

[14] Gesing A, Lewiński A, Karbownik-Lewińska M (2012). The thyroid gland and the process of aging; what is new?. Thyroid Res..

[15] Ichiki T (2016). Thyroid hormone and vascular remodeling. J. Atheroscler. Thromb..

[16] Prisant LM, Gujral JS, Mulloy AL (2006). Hyperthyroidism: a secondary cause of isolated systolic hypertension. J. Clin. Hypertens. (Greenwich)..

[17] Napoli R (2001). Impact of hyperthyroidism and its correction on vascular reactivity in humans. Circulation.

[18] Ojamaa K, Klemperer JD, Klein I (1996). Acute effects of thyroid hormone on vascular smooth muscle. Thyroid.

[19] Danzi S, Klein I (2003). Thyroid hormone and blood pressure regulation. Curr. Hypertens. Rep..

[20] Shimizu Y (2019). Reticulocyte levels have an ambivalent association with hypertension and atherosclerosis in the elderly: a cross-sectional study. Clin. Interv. Aging..

[21] Shimizu Y (2020). Insulin-like growth factor-1 (IGF-1) and reduced tongue pressure in relation to atherosclerosis among community-dwelling elderly Japanese men: A cross-sectional study. Dysphagia.

